# Preferential Attraction of Oviposition-Ready Oriental Fruit Flies to Host Fruit Odor over Protein Food Odor

**DOI:** 10.3390/insects12100909

**Published:** 2021-10-06

**Authors:** Gwang-Hyun Roh, Paul E. Kendra, Dong H. Cha

**Affiliations:** 1 USDA-ARS, US Pacific Basin Agricultural Research Center, Hilo, HI 96720, USA; roh.gwanghyun82@gmail.com; 2 Oak Ridge Institute for Science and Education, Oak Ridge, TN 37830, USA; 3 USDA-ARS, Subtropical Horticulture Research Station, Miami, FL 33158, USA; paul.kendra@usda.gov

**Keywords:** egg load, female attraction, food choice, host choice, invasive pest, physiological status

## Abstract

**Simple Summary:**

Oriental fruit fly, *Bactrocera dorsalis*, is one of the most destructive invasive pests of tropical fruit and vegetable crops worldwide. Current oriental fruit fly quarantine programs focus heavily on the control and surveillance of male flies, which is less effective for mitigating the impact caused by female populations. We investigated the relationship between olfactory preference and oviposition outcome of oriental fruit flies. In laboratory bioassays using similarly aged (14–16 day old) cohorts of mated females, some females preferred host fruit odors over protein food odor (torula yeast), while some preferred protein odor. The females that preferred host fruit odor had 2.1 times greater egg load and laid 2.4 times more eggs than females that preferred protein odor. Our results suggest that mated female oriental fruit flies with a preference for host fruit odor are likely to be in an imminent oviposition-ready physiological status, while mated females that prefer torula yeast odor are likely more protein-hungry and need more protein to produce the critical egg load necessary for oviposition.

**Abstract:**

Olfaction plays a key role in the location of food and oviposition resources by tephritid fruit flies. Adult females, including oriental fruit fly, *Bactrocera dorsalis*, can sustain egg production throughout their lives provided they obtain sufficient protein. Thus, preferential attraction to food or oviposition sites (host fruit) will depend on a fly’s particular physiological state. In this study, laboratory bioassays were conducted with mature, mated *B. dorsalis* (provisioned protein and sugar *ad libitum*) to evaluate attraction to traps baited with torula yeast versus six host fruit sources (guava, guava juice, mango, orange, Surinam cherry, or white sapote). Females that preferred fruit laid a significant number of eggs around the trap entrance (average 405 eggs/fly), while almost no eggs were laid by females that preferred yeast (0.5 and 1.3 eggs/fly on two occasions). Similar results were observed in a bioassay using headspace extracts of guava juice and torula yeast, supporting olfactory-mediated responses. When individual females were allowed to oviposit in guava juice traps 0–24 h after a choice test, 45.8% of females that chose guava juice laid eggs (average 14.7 eggs/fly), compared with 27.5% that chose yeast (average 6.5 eggs/fly). Dissections indicated that females with a preference for guava juice had an egg load 2.4 times greater than females that preferred yeast. These results suggest there is an olfactory-based behavioral switch in preference from protein to host odors when female *B. dorsalis* are oviposition-ready. We discuss the implications of our findings for practical behavioral management and detection programs for *B. dorsalis*.

## 1. Introduction

Oriental fruit fly, *Bactrocera dorsalis* (Hendel) (Diptera: Tephritidae), is one of the most destructive invasive pests of tropical fruit and vegetable crops worldwide, with more than 430 hosts, including many important economic crops. Consequently, it poses a serious international trade barrier [1,2]. Although established in Hawaii since 1964 [3], *B. dorsalis* has not established on the U.S. mainland despite repeated incursions since its first detection in 1960 in California; however, both the frequency and size of *B. dorsalis* incursions have been accelerating. This escalates the potential for catastrophic trade disruption and reduced availability and marketability of produce, as witnessed from the 2018 *B. dorsalis* outbreak in Florida [4]. Current *B. dorsalis* quarantine programs focus heavily on control and surveillance of male flies using combinations of sterile insect technique, male annihilation technique, and extensive networks of male monitoring traps [5,6]. However, this male-oriented strategy is less effective for mitigating the impact caused by female populations, which could lead to fruit damage and ensuing trade issues.

Developing effective behavioral-based methods for the detection and control of female fruit flies has been a research priority for fruit fly management programs [7]. Protein odor and host fruit odor are two classes of volatile cues used by female fruit flies to locate protein-rich food sources and oviposition sites, respectively [8], and thus may be exploited as attractants [9,10,11,12,13]. It has been suggested that physiological status (e.g., mating status, age, and hunger level) has a strong influence on food and host choice decisions made by female fruit flies [14], as they may be preferentially attracted to the odor cues that are released from resources that most satisfy their needs at a given moment [15]. Thus, an unmated female fruit fly is considered more responsive to protein-based odors, while a mated, sexually mature female fly may be more interested in host fruit-based odors [14,16]. This is supported by electroantennography studies with the Caribbean fruit fly, *Anastrepha suspensa* (Loew), which demonstrated that sexually immature females have a higher olfactory response to ammonia (a protein cue), whereas mature gravid females have a higher olfactory response to CO_2_ (a short-range oviposition cue) [17,18]. In fact, extensive research has been conducted on a variety of food-based and host plant-based fruit fly attractants for the development of field lures (reviewed in [19,20]). In particular, for *B. dorsalis*, liquid protein-based baits, such as hydrolyzed torula yeast, have been commercially available and widely used as a female-biased attractant in management programs, although inconsistencies in trap performance have been a concern [21,22].

In terms of behavioral management, it is likely that a particular lure is attractive to only a fraction of a fruit fly population at any given time and space, as flies in an area may vary widely in age structure and physiological status [15]. Knowing what subset of females a lure can attract—i.e., understanding how physiological status influences olfactory chemoreception and female preference for food versus host fruit odors—and to what extent the preferential attraction affects fruit infestation may inform fruit fly management decisions. For example, both effective protein-based and host-based lures may be able to reduce fruit infestation, but by targeting females from different classes of physiological status. Studies have shown that a protein odor-based lure may be more effective for capture of unmated females as compared with mated ones, protein-hungry females as compared with protein-fed females, or nongravid females as compared with oviposition-ready flies; and vice versa with host fruit odor-based lures [11,16,23,24,25].

Although it has been suggested that female flies become more responsive to host fruit odors than to protein food odors after mating, even mated females benefit from sustained feeding on proteins, hence attraction to them, until they become oviposition-ready [26,27]. Moreover, tephritid fruit flies can produce eggs throughout their adult period as long as they have access to protein food [28]. Therefore, females will continue to search for protein sources to supply the resources required for ovarian development and vitellogenesis [26,29]. In *Bactrocera* fruit flies, egg load was the most important factor that determined the behavioral switch from resource foraging to host acceptance and oviposition [30], with a significant association between continuous protein feeding and a greater degree of adult ovary development, egg load, and oviposition rate [27].

Here, we investigated the attraction of gravid *B. dorsalis* females to protein odors and host fruit odors and determined their oviposition outcome based on odor choice. All tests used mature (14–16-day-old) male and female *B. dorsalis* that were provided *ad libitum* access to a protein and sugar diet and mated freely after emergence from pupae. In the first set of experiments, we (i) evaluated the odor preference of mated *B. dorsalis* females between torula yeast and six host fruits using laboratory two-choice cage experiments, (ii) determined the most attractive host fruit source using a multiple-choice experiment, and (iii) confirmed that the *B. dorsalis* attraction was mediated by olfactory cues. In the second set of experiments, we challenged individual mated females for oviposition in response to a host fruit odor immediately after they exhibited preferential attraction to an odor source in two-choice tests, and subsequently evaluated the number of eggs laid and the internal egg load based on the odor preference of each female.

## 2. Materials and Methods

### 2.1. Insects

*Bactrocera dorsalis* were reared at the fruit fly rearing facility at the USDA ARS Pacific Basin Agricultural Research Center in Hilo, Hawaii. Larvae were reared on a standard wheat, sugar, and yeast diet [31]. Cohorts of flies eclosed from pupae (total 12 different generations used) were grouped over two days and held in screened cages (30 cm W × 30 cm L × 30 cm H; shop.bugdorm.com) for two weeks at 24 °C, 60–80% RH, 12:12 L/D on unlimited water, sugar, and hydrolyzed yeast, and mated freely prior to testing. Three hours prior to behavioral assays, female and male flies (14–16 days old) were separated and counted in a 5 °C walk-in refrigerator [32]. These flies were considered mated as >95% of females were mated by day 7 under similar conditions [32] and all of the 14–16-day-old females that we dissected had developed eggs.

### 2.2. Host Fruits and Torula Yeast

The test material consisted of five known host fruits of *B. dorsalis*, guava juice, and torula yeast. Locally produced mango (*Mangifera indica*; unknown variety) and orange (*Citrus sinensis*; navel) were purchased from local groceries. Common guava (*Psidium guajava*), Surinam cherry (*Eugenia uniflora*), and white sapote (*Casimiroa edulis*) were collected from fruit orchards in Hilo and Kona, Hawaii. All fruits were tested at ripe stage and washed in water to remove any residue. Guava juice (Meadow Gold, Hilo, HI, USA) was purchased from local groceries. Torula yeast/borax pellets were purchased from Better World Manufacturing, Inc. (Fresno, CA, USA).

### 2.3. Attraction and Oviposition Response Bioassay

Behavioral assays were conducted using a gated cup trap, an aluminum foil-covered 100 mL or 250 mL glass beaker with a cut centrifuge vial (0.7 cm diameter) inserted in the center of the foil, which restricted adult flies from escaping once they enter the trap [33]. In attraction bioassays, gated cup traps were baited with one of the following: (1) a 5 g piece of host fruit (guava, mango, orange, Surinam cherry, or white sapote) +30 mL of 0.0125% soapy water (unscented soap: Colgate-Palmolive Company, New York, NY, USA; used as drowning solution [33]), (2) 30 mL of guava juice, (3) 30 mL of torula yeast solution (5 g torula yeast/30 mL water), (4) a rubber septum loaded with 200 µL guava juice headspace extract (see below) +30 mL of 0.0125% soapy water, or (5) a rubber septum loaded with 200 µL torula yeast headspace extract +30 mL of 0.0125% soapy water. Gated cup traps were also used to evaluate the oviposition response from female *B. dorsalis* attracted to a trap baited with different odor sources (e.g., host fruit, torula yeast). Females exhibited oviposition behavior by inserting their ovipositor into the gap between aluminum foil and cut centrifuge vial.

### 2.4. Comparison of Mated B. dorsalis Attraction and Oviposition Response to Host Fruit versus Torula Yeast

We compared the attractiveness of a host fruit source (guava, guava juice, mango, orange, Surinam cherry, or white sapote) versus torula yeast in 30 cm × 30 cm × 30 cm screened cages (shop.bugdorm.com) using a two-choice assay (N = 4). Each cage was provided with two 100 mL gated cup traps—one baited with a host fruit source and one baited with torula yeast (see above), placed 15 cm apart—and a wet cotton. Mated *B. dorsalis* (50 females and 50 males) were released in each cage. After 24 h, the number of male and female flies captured and number of eggs oviposited in gated cup traps were counted. When less than 100 eggs were oviposited, eggs were counted individually under a microscope. When more than 100 eggs were oviposited, eggs were counted volumetrically using the conversion of 1 mL eggs in water = 20,000 eggs (Stephanie Gayle, personal communication).

### 2.5. Selection of a Preferred Host Fruit Using Multiple-Choice Test

The multiple-choice test (N = 9) was conducted using 60 cm × 60 cm × 60 cm screened cages (shop.bugdorm.com) as described in [34]. Briefly, within each cage, seven 250 mL gated cup traps (six traps baited with one of guava, guava juice, mango, orange, Surinam cherry, and white sapote, and one trap baited with torula yeast) were randomly positioned in a circle (55 cm diameter) with equal distance (20 cm apart) from each trap along the circumference. Mated *B. dorsalis* (100 males and 100 females) were released in each cage. The number of male and female flies captured inside each trap was counted after 24 h.

### 2.6. Headspace Volatile Collection

To confirm whether the behavioral responses from mated *B. dorsalis* observed in the above experiments were mediated by odors of host fruit and torula yeast, headspace volatiles were collected from guava juice or torula yeast. Headspace volatiles released from the same amount of guava juice (30 mL) or torula yeast (5 g dissolved in 30 mL water) used in attraction and oviposition bioassays were collected using 2.4 L closed volatile collection chambers (ARS, Inc., Gainesville, FL, USA) with one air inlet adapter (7 mm ID) on the top and an outlet adapter (7 mm ID) on the bottom wall. Clean air was drawn into each collection chamber through an activated coconut charcoal filter, and then drawn out (0.7 L/min) through a charcoal adsorbent trap (ORBO32, Supelco Inc., Bellefonte, PA, USA) over 24 h. Headspace volatile compounds were eluted from the adsorbent trap by rinsing with 750 μL methylene chloride. The extracts were stored at −20 °C until use.

### 2.7. Comparison of Mated B. dorsalis Attraction and Oviposition Response to Headspace Extracts of Host Fruit versus Torula Yeast

The attraction and oviposition responses of mated *B. dorsalis* adults to headspace extracts of guava juice and torula yeast were compared using the same two-choice bioassay design described above in 30 cm × 30 cm × 30 cm screened cages (N = 4). For treatments, 200 µL of guava juice headspace extract or torula yeast headspace extract was loaded on rubber septa (Chemglass Life Science, Vineland, NJ, USA). The septum sources were prepared 60 min prior to a test. In each cage, two gated cup traps, each containing a rubber septum loaded with guava juice extract or rubber septum loaded with torula yeast extract along with 30 mL of 0.0125% soapy water, were provided. Mated *B. dorsalis* (50 males and 50 females) were released into each cage. The number of flies and number of eggs oviposited in each trap were counted after 24 h.

### 2.8. Oviposition Response of Mated B. dorsalis Attracted to Guava Juice versus Torula Yeast

In this experiment, we compared the oviposition response and egg load from individual mated female *B. dorsalis* preferentially attracted to either host fruit odor or protein odor. To select flies with a preference for host fruit odor or protein odor, we placed two 250 mL gated cup traps—one baited with 30 mL of guava juice and one baited with 5 g of torula yeast +30 mL water—in a 30 cm × 30 cm × 30 cm screened cage, released 30 mated *B. dorsalis* females, and live-captured individual flies as soon as they entered a trap. To facilitate the collection of live females, we inserted a mesh screen inside the trap to prevent flies from drowning inside traps.

Individual flies that chose either guava juice odor or torula yeast odor were immediately re-released into 30 cm × 30 cm × 30 cm screened cages (one fly/cage) with a 250 mL “modified” gated cup trap baited with 30 mL of guava juice and allowed to oviposit over 24 h (N = 51). The modified gated traps were identical to the standard gate cup trap described above, but with the entrance to the trap (i.e., cut centrifuge vial) plugged with a piece of cotton to prevent female flies from entering the trap. The number of eggs oviposited by individual flies was monitored over three different time intervals, between 0 and 3 h, 3 and 6 h, and 6 and 24 h after fly introduction to the oviposition cage. Gated traps in each cage were replaced with a new modified gated trap at 3 h and 6 h post fly release into the cage. Th percentage of flies that oviposited was also calculated.

Egg load was also determined for individual mated *B. dorsalis* females that preferentially chose either guava juice odor or torula yeast odor (N = 20 for each). Immediately after they were captured in either guava juice or torula yeast baited traps, individual flies were released into different screened cages (1 fly/cage) provided with water (wet cotton) only. The abdomen of each fly was then dissected under a microscope and the number of eggs was counted (e.g., [35]).

### 2.9. Statistical Analyses

Trap capture, number of eggs oviposited, and egg load data were analyzed using a generalized linear mixed model in a randomized block design with replicate as a random factor and different bait treatments as a fixed factor using Poisson distribution with log link function and maximum likelihood estimation. The means were compared using the Tukey–Kramer test (Proc Glimmix, [36]).

## 3. Results

### 3.1. Comparison of Mated B. dorsalis Attraction and Oviposition Response to Host Fruit versus Torula Yeast

**Figure 1:**
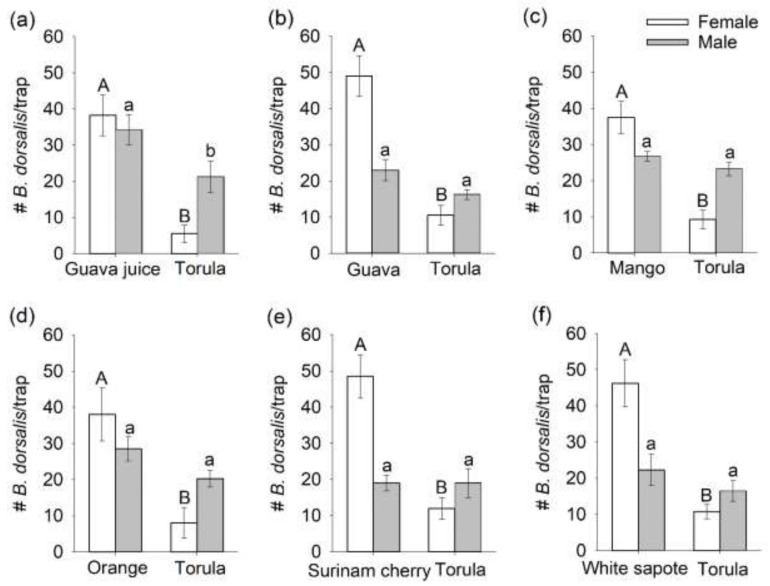
Mean (±SEM) numbers of mated male and female *Bactrocera dorsalis* flies captured in traps baited with torula yeast and one of the six host fruit sources ((**a**) guava juice, (**b**) guava, (**c**) mango, (**d**) orange, (**e**) Surinam cherry, and (**f**) white sapote) in two-choice laboratory assays. For a given sex, different letters on bars indicate significant differences by Tukey–Kramer tests at *p* < 0.05.

Significantly more mated females were captured in traps baited with any of the six host fruit sources than in traps baited with torula yeast (mango: F_1,3_ = 58.15, *p* = 0.0047; orange: F_1,3_ = 64.18, *p* = 0.0041; guava juice: F_1,3_ = 72.35, *p* = 0.0034; guava: F_1,3_ = 82.08, *p* = 0.0028; white sapote: F_1,3_ = 74.29, *p* = 0.0033; Surinam cherry, F_1,3_ = 75.06, *p* = 0.0032; Figure 1).

**Figure 2:**
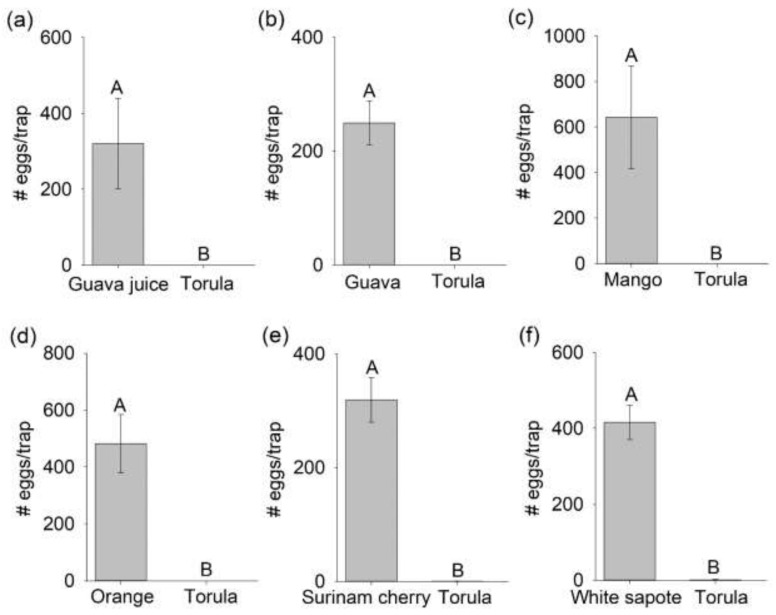
Number of *Bactrocera dorsalis* eggs (mean ± SEM) oviposited in gated cup traps baited with either torula yeast and one of the six host fruit sources ((**a**) guava juice, (**b**) guava, (**c**) mango, (**d**) orange, (**e**) Surinam cherry, and (**f**) white sapote) in two-choice laboratory assays. Different letters on bars indicate significant differences by Tukey–Kramer tests at *p* < 0.05.

In contrast, there were no significant differences in the number of male flies captured in traps baited with a host fruit source or torula yeast (all *p*-values > 0.1000), except for guava juice baited traps, which captured significantly more males than torula yeast baited traps (F_1,3_ = 11.95, *p* = 0.0407). Female flies attracted to a host fruit source oviposited a significantly greater number of eggs (>200 eggs/trap) than females attracted to torula yeast (mango: F_1,3_ = 61.64, *p* = 0.0043; orange: F_1,3_ = 57.18, *p* = 0.0048; guava juice: F_1,3_ = 51.16, *p* = 0.0056; guava: F_1,3_ = 47.66, *p* = 0.0062; white sapote: F_1,3_ = 168.13, *p* = 0.0001; Surinam cherry, F_1,3_ = 83.31, *p* = 0.0028; Figure 2). A total of only seven eggs were oviposited in traps baited with torula yeast (two eggs in one torula yeast trap in the Surinam cherry bioassay and five eggs in a torula yeast trap in the white sapote assay), even though 41% of females chose torula yeast baited traps in two-choice assays.

### 3.2. Selection of a Preferred Host Using Multiple-Choice Test

**Figure 3:**
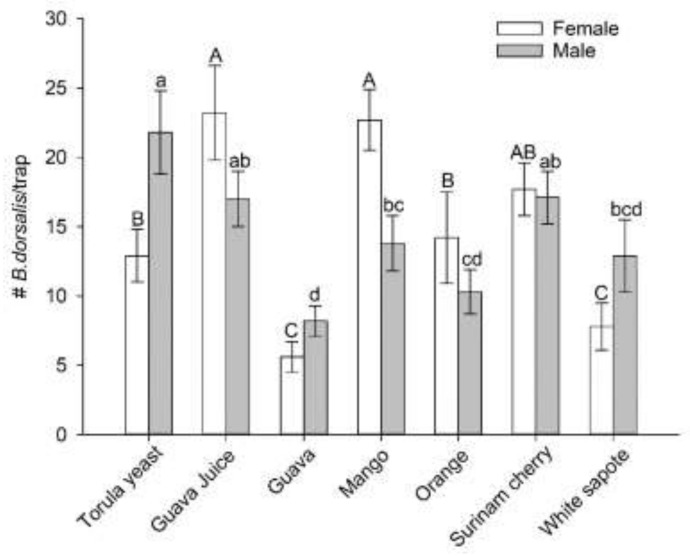
Mean (±SEM) numbers of mated male and female *Bactrocera dorsalis* flies captured in traps baited with torula yeast and six different host fruit sources in multiple-choice laboratory assays. For a given sex, different letters on bars indicate significant differences by Tukey–Kramer tests at *p* < 0.05.

When traps baited with guava, guava juice, mango, orange, Surinam cherry, and white sapote were compared simultaneously, traps baited with either guava juice or mango captured significantly greater numbers of female flies than traps baited with torula yeast; however, the greatest number of females was captured in traps baited with guava juice (F_6,48_ = 25.73, *p* < 0.0001, Figure 3). For male flies, traps baited with torula yeast captured the greatest number of flies and there were no significant differences among numbers captured in traps baited with torula yeast, guava juice, or Surinam cherry (F_6,48_ = 12.59, *p* < 0.0001, Figure 3).

### 3.3. Comparison of Mated B. dorsalis Attraction and Oviposition Response to Headspace Extracts of Host Fruit versus Torula Yeast

**Figure 4:**
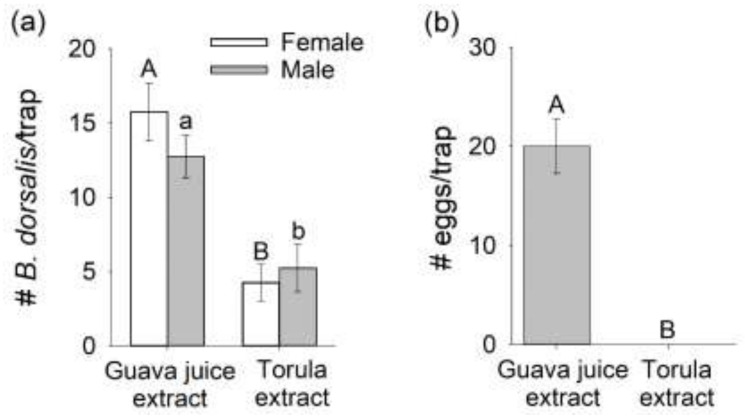
Mean (±SEM) numbers of (**a**) mated male and female *Bactrocera dorsalis* flies captured and (**b**) eggs oviposited in traps baited with headspace extracts of guava juice and torula yeast in two-choice laboratory assays. For a given sex, different letters on bars indicate significant differences by Tukey–Kramer tests at *p* < 0.05.

Attraction and oviposition response of mated *B. dorsalis* to guava juice and torula yeast odors released from rubber septa (Figure 4) were similar to that observed with the actual guava juice and torula yeast in the previous experiment (Figure 1 and Figure 2). Traps baited with guava juice headspace extract captured significantly greater numbers of male and female flies than traps baited with torula yeast headspace extract (female, F_1,3_ = 22.97, *p* = 0.0173, male, F_1,3_ = 11.71, *p* = 0.0418, Figure 4a). In addition, mated females oviposited a significantly greater number of eggs in traps baited with guava juice headspace extract than in traps baited with torula yeast headspace extract (zero eggs in torula yeast baited traps: F_1,3_ = 18.97, *p* = 0.0224; Figure 4b).

### 3.4. Oviposition Response from Mated B. dorsalis Preferentially Attracted to Guava Juice versus Torula Yeast

**Figure 5:**
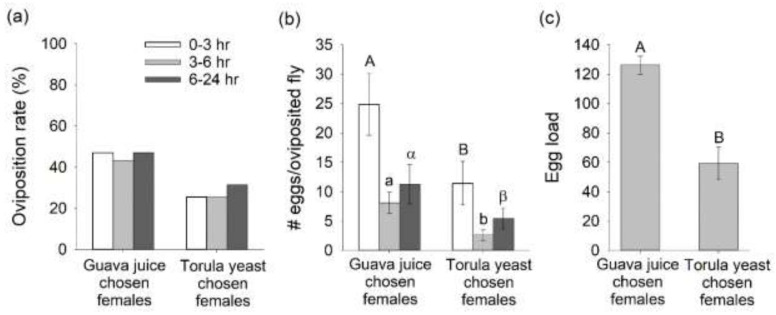
Oviposition responses (**a**,**b**) and egg load (**c**) from mated female *Bactrocera* *dorsalis* immediately after individual female flies made a choice between guava juice or torula yeast. Oviposition responses were evaluated at 0–3, 3–6, and 6–24 h post-choice period. Different letters indicate significant difference between guava juice and torula yeast by Tukey–Kramer tests at *p* < 0.05.

When individual mated *B. dorsalis* females that chose guava juice or torula yeast were challenged to oviposit in response to guava juice odor over 24 h, the oviposition rates (%) from flies that preferred guava juice were 85, 69, and 50% greater than oviposition rates from females that chose torula yeast during the 0–3, 3–6, and 6–24 h post-choice period, respectively (Figure 5a). Comparing females that chose guava juice or torula yeast odor and successfully oviposited, the average number of eggs laid by females that chose guava juice was 2.2, 3.1, and 2.1 times greater than that laid by females that chose torula yeast during the 0–3, 3–6, and 6–24 h post-choice time intervals, respectively (0–3 h: F_1,98_ = 240.8, *p* < 0.0001; 3–6 h: F_1,98_ = 129.16, *p* < 0.0001; 6–24 h: F_1,98_ = 98.77, *p* < 0.0001; Figure 5b). The response of mated *B. dorsalis* females to host fruit odor and torula yeast odor appeared to be related to egg load. The average egg load of females that preferred guava juice odor was 2.1 times greater than the egg load of flies that preferred torula yeast odor (F_1,19_ = 467.97, *p* < 0.0001; Figure 5c).

## 4. Discussion

Our results show that considerable variation exists for olfactory preference and oviposition outcome among the cohorts of 14–16-day-old mated *B. dorsalis* females fed an unlimited diet of sugar, hydrolyzed protein, and water. Although a significantly greater number of mated females preferred the odors emitted from host fruit over the odor from torula yeast, about 41% of the females were still preferentially attracted to the odor from torula yeast. Mated females that differed in odor preference also exhibited significant differences in egg load and oviposition outcome. Females that preferred host fruit odor had an egg load 405 times greater than females that preferred torula yeast odor. When these flies were challenged to oviposit immediately after they made a choice between host fruit and protein odors, 45.8% of the female *B. dorsalis* that chose host fruit odor laid eggs during the 24 h post-choice period, while only 27.5% of females that chose torula yeast laid eggs. Moreover, among those flies that did oviposit, females attracted to host fruit odor laid 2.4 times more eggs than females attracted to torula yeast. Together, these results suggest that mated female *B. dorsalis* with a preference for host fruit odor are likely to be in an imminent oviposition-ready physiological status, while mated females that prefer torula yeast odor are likely more protein-hungry and need to ingest more protein to build up a critical egg load necessary for oviposition [23,27,28,37,38,39].

The findings on differential oviposition outcomes from gravid *B. dorsalis* with differential olfactory preferences are in agreement with previous studies. These studies suggest that (1) tephritid fruit flies show compensatory feeding patterns on limited resources at a given moment (e.g., protein deprived flies are more interested in ingesting protein than other resources such as sugar; [40]); (2) younger flies are more attracted to a protein source than host fruit as protein ingestion is necessary to become reproductively active [17,24,26,27,29,41,42]; (3) mated and older females become more attracted to host fruit than protein food, which is related to host acceptance and oviposition [11,16,17,24,25,30,43,44]; (4) fruit flies with a greater egg load have a greater oviposition outcome [27]; and (5) fruit flies with a greater amount of protein ingestion have a greater egg load [45]. Our results add to these studies and provide data that even similarly aged mated cohorts of female *B. dorsalis*, with the same diet history, can exhibit differential olfactory preferences for protein food and host fruit odors, preferences that are dependent on female physiological status and have a direct impact on their oviposition outcome. We recognize that our findings are based on 14–16-day-old females. In the field, the temporal distribution of females ready for oviposition is often unknown. Thus, future studies will evaluate whether similar outcomes are observed from females with a wider age distribution.

From a management perspective, although it has been generally suggested that a protein-based attractant is more effective for the capture of young unmated female *B. dorsalis,* while a host fruit-based attractant is more effective for mated females [11,13,25,43,46], our results strongly suggest that a protein-based attractant should still be effective for the capture of mated, but less oviposition-ready female *B. dorsalis*. Moreover, the proportion of these protein-hungry females is likely even greater under field conditions, considering that protein is a limited resource in habitats of tropical fruit flies [47] and that *B. dorsalis* tested in our study had unlimited access to protein and sugar as adults and larvae. This suggests that an effective protein-based attractant may be attractive to a larger proportion of fruit flies in the field than previously predicted [48].

Our results also support the potential usefulness of a host-based attractant in female *B. dorsalis* management programs. The current study documented a clear preference for host fruit odors by oviposition-ready *B. dorsalis* females, the proportion of the pest population that poses a direct threat to fruit production. In tandem with an effective repellent applied to fruit trees, a perimeter of traps baited with a host-based lure can be deployed using a ‘push-pull’ strategy to reduce the number of oviposition-ready females [49]. Alternatively, an array of traps combining host-based and protein-based lures may improve detection and suppression efforts by targeting females of different physiological status. With *A. suspensa*, there is synchronous maturation of eggs within ovarioles, resulting in cyclical increases in the egg load and oviposition of ‘batches’ of eggs [26]. Therefore, when a female has a mature batch of eggs to lay, she will be more attracted to host odors; however, once the batch is laid, she will switch preference to protein odors to complete maturation of the next batch of eggs. Together, these results suggest that both protein-based and host fruit-based attractants may play valuable roles in reducing fruit infestation by targeting *B. dorsalis* females with differential physiological needs.

Protein baits such as torula yeast solutions have been used as an attractant for female *B. dorsalis* [4,21]. The bait is effective for both male and female flies, but is considered to be a weak attractant, especially compared with strong male lures such as methyl eugenol [50]. Liquid protein baits are also difficult to maintain and service, not user friendly, not specific, and known for inconsistencies in effectiveness [51,52,53,54]. The inconsistencies in trap attractiveness may be related to fluctuations in protein bait odor profiles, which are affected by biotic (e.g., microbial growth) and abiotic conditions surrounding the trap [19]. Understanding the key essential protein bait odor compounds that are directly responsible for fruit fly attraction may help overcome the inconsistency. Work is currently in progress to identify the key attractant compounds released from the headspace of protein baits preferred by female *B. dorsalis* to develop more consistent and user-friendly female lures that can replace protein baits. Practical application of host fruit odor-based attractants in the field is still limited for *B. dorsalis* [55], although there have been studies to identify host-based attractants [13,43,46]. The results from the multiple-choice assay suggest that guava juice and other fruit volatiles could be a good source for the development of a synthetic attractant for *B. dorsalis* females, especially close to oviposition. Work is under way to develop an attractive female lure particularly effective for oviposition-ready *B. dorsalis* in the field and to test whether capturing oviposition-ready *B. dorsalis* actually results in a reduction in fruit infestation.
